# Glucosamine Interferes With Myelopoiesis and Enhances the Immunosuppressive Activity of Myeloid-Derived Suppressor Cells

**DOI:** 10.3389/fnut.2021.762363

**Published:** 2021-11-10

**Authors:** Eric Chang-Yi Lin, Shuoh-Wen Chen, Luen-Kui Chen, Ting-An Lin, Yu-Xuan Wu, Chi-Chang Juan, Yuan-I Chang

**Affiliations:** ^1^Department and Institute of Physiology, College of Medicine, National Yang Ming Chiao Tung University, Taipei City, Taiwan; ^2^Department of Internal Medicine, School of Medicine, College of Medicine, National Yang Ming Chiao Tung University, Taipei City, Taiwan; ^3^Division of Hematology and Oncology, Department of Medicine, Taipei Veterans General Hospital, Taipei City, Taiwan

**Keywords:** d-Glucosamine hydrochloride (PubChem CID: 91431), hematopoietic stem cell (HSC), myelopoiesis, myeloid-derived suppressive cell, immunosuppression

## Abstract

Glucosamine (GlcN) is the most widely consumed dietary supplement and exhibits anti-inflammatory effects. However, the influence of GlcN on immune cell generation and function is largely unclear. In this study, GlcN was delivered into mice to examine its biological function in hematopoiesis. We found that GlcN promoted the production of immature myeloid cells, known as myeloid-derived suppressor cells (MDSCs), both *in vivo* and *in vitro*. Additionally, GlcN upregulated the expression of glucose transporter 1 in hematopoietic stem and progenitor cells (HSPCs), influenced HSPC functions, and downregulated key genes involved in myelopoiesis. Furthermore, GlcN increased the expression of arginase 1 and inducible nitric oxide synthase to produce high levels of reactive oxygen species, which was regulated by the STAT3 and ERK1/2 pathways, to increase the immunosuppressive ability of MDSCs. We revealed a novel role for GlcN in myelopoiesis and MDSC activity involving a potential link between GlcN and immune system, as well as the new therapeutic benefit.

## Introduction

Glucosamine (GlcN; 2-amino-2-deoxy-D-glucose) is an essential aminomonosaccharide synthesized from glucose and is utilized for biosynthesis of glycosylated proteins and lipids. GlcN is present in almost all tissues, with the highest concentrations found in the cartilage. As GlcN functions in cartilage and synovial fluid synthesis, GlcN has become one of the most widely consumed dietary supplement globally as a nutraceutical for chondroprotection (1, 2). Binding to the transmembranous transport is the first step for GlcN uptake. The glucose transporter (GLUT) 1, 2 and 4 facilitate GlcN transportation in *Xenopus* oocytes (3). In chondrocytes, GlcN treatment increases plasma membrane expression of GLUT1 and 6 (4). Numerous studies have demonstrated the potential of GlcN for treating osteoarthritis mainly because of its chondroprotection and anti-inflammatory effects (5–8). In addition, new biological and pharmacological applications of GlcN in bacterial infection, cancers, cardiovascular diseases have been reported. Studies have suggested that GlcN exerts most of its functions by suppressing inflammatory pathways and decreasing pro-inflammatory cytokine production and enzyme expression. Notably, GlcN is a promising candidate for the prevention and/or treatment of other diseases because of its anti-oxidant and anti-inflammatory activities (7, 9). Limited evidence has indicated that GlcN modulates T cell differentiation (10); however, whether GlcN influences the function of hematopoietic stem and progenitor cells (HSPCs) and myelopoiesis is not well-understood.

Myeloid cells are the direct effectors of innate immunity and promote adaptive immune responses (11). However, conditions involving chronic inflammation, such as autoimmune diseases and cancers, cause the aberrant generation and expansion of myeloid cells that are phenotypically and functionally distinct from normal myeloid cells and facilitate, rather than cease, disease progression. Among these myeloid regulatory cells, myeloid-derived suppressor cells (MDSCs) are key myeloid regulatory subtypes (12). MDSCs are a heterogeneous population of myeloid progenitor cells and immature myeloid cells, and exhibit a remarkable ability to suppress T-cell responses (11). MDSCs were first characterized in cancers, where they were shown to promote cancer progression and metastasis through immunosuppression of T cells (13). Recent studies highlighted their immune regulatory functions in various disease settings, such as viral infections (14, 15), some autoimmune diseases, and graft-vs.-host disease (16). Moreover, clinical data have suggested MDSCs exert anti-inflammatory effects during the acute inflammatory phase in patients with COVID-19 (17).

The suppressive activity of MDSCs is highly associated with L-arginine metabolism. L-Arginine is the substrate of two enzymes, inducible nitric oxide synthase (iNOS, which generates nitric oxide, NO) and arginase-1 (Arg-1, which converts L-arginine to urea and L-ornithine). MDSCs express high levels of these enzymes. Higher Arg-1 activity in MDSCs results in higher L-arginine catabolism that leads to the depletion of this non-essential amino acid from the microenvironment, resulting in the suppression of T-cell proliferation. MDSCs also produce NO by expressing iNOS to downregulate T cell function. Another important factor contributing to the suppressive activity of MDSCs is reactive oxygen species (ROS). Inhibition of ROS production by MDSCs completely abrogated the suppressive effect of these cells (11, 18, 19). Given that GlcN mediates an anti-inflammation effects in various pathophysiological processes, we hypothesized that GlcN can reprogram myelopoiesis to interfere with immune cell production. In this study, we examined the role of GlcN in myelopoiesis and MDSC production as well as the mechanisms of the MDSC-mediated suppressive effect.

## Materials and Methods

### Mice

The 8–12 week-old male C57BL/6 mice were obtained from the Laboratory Animal Center of National Yang Ming Chiao Tung University (Taipei Yang Ming Campus). D-(+)-Glucosamine hydrochloride (purity 99.9%) was obtained from Sigma-Aldrich (St. Louis, MO, USA). D-(+)-Glucosamine hydrochloride dissolved in phosphate-buffered saline (PBS) was administered via an intraperitoneal (i.p.) injection (30 mg/mouse/day) into the mice for 14 days according to a previous report (20) or implanted subcutaneously using Alzet model 1002 osmotic pumps (1 M, 0.25 μL/h) (DURECT Corp., Cupertino, CA, USA) to maintain a steady GlcN concentration. After 14-day of GlcN treatment, mice were sacrificed with carbon dioxide inhalation. Mice treated with PBS were used as control. Complete blood count analysis was performed on a XN-450 hematology analyser (Sysmex, Kobe, Hyogo, Japan). All animal experiments were conducted in accordance with the Guide for the Care and Use of Laboratory Animals and approved by the Institutional Animal Care and Use Committee (IACUC) of National Yang Ming Chiao Tung University (Taipei Yang Ming campus) (IACUC number: 1081005).

### Bone Marrow and Spleen Cell Isolation

Bone marrow (BM) cells were carefully isolated from the tibia and femur. For spleen (SP) cell preparation, a small piece of excised SP was pressed through a strainer using the plunger end of a syringe. Subsequently, BM and SP cells were washed through the strainer and collected in PBS containing 2% fetal bovine serum (FBS), which was obtained from Gibco (Grand Island, NY, USA), for immediate use (21, 22).

### Flow Cytometric Analysis and Cell Sorting

For hematopoietic cell analysis, flow cytometric analyses were performed as previously described (23). Briefly, antibodies specific for the following surface antigens were purchased from BD Biosciences (Franklin Lakes, NJ, USA): B220 (RA3-6B2), CD3 (145-2C11), CD4 (GK1.5), CD8 (53-6.7), CD11b (M1/70), CD34 (RAM34), CD41 (MWReg30), CD45 (30-F11), CD48 (HM48-1), CD150 (Q38-480), FcγRIII/FcγRII (2.4G2), Gr-1 (RB6-8C5), IgM (R6-60.2), IL-7Ra (B12-1), c-Kit (2B8), Ly-6C (AL-21), Ly-6G (1A8), Sca-1 (D7), streptavidin and Ter119 (TER-119). CD19 (1D3) and Thy1.2 (53-2.1) were purchased from eBioscience (San Diego, CA, USA) (The detail antibody panels for detecting different hematopoietic cells can be found in Supplementary Tables 1–4). For detection of reactive oxygen species, cells were stained with 5 μM 2′,7′-dichlorofluorescin diacetate (Merck, Darmstadt, Germany). Flow cytometry analyses were performed on a CytoFLEX S flow cytometer (Beckman Coulter, Brea, CA, USA) and results were analyzed with FlowJo software (TreeStar, Ashland, OR, USA).

For isolating Lin^−^c-Kit^+^Sca-1^+^ (LSK) cell population, which expresses Sca-1 and c-Kit but lacks the lineage (Lin) markers expressed on mature myeloid and lymphoid cells, myeloid progenitors (MP, Lin^−^c-Kit^+^Sca-1^−^) and granulocyte-macrophage progenitors (GMP, Lin^−^c-Kit^+^Sca-1^−^CD34^+^FcRII/III^+^), BM were extracted and stained with antibodies (Supplementary Table 2). The c-Kit^+^ cells were enriched firstly by using anti-allophycocyanin (APC) Magnetic Particles (E30-221), which was obtained from BD Bioscience. Then, LSK, MP, and GMP cells were sorted from the c-Kit^+^ cell fraction using BD FACS Melody cell sorter (BD Biosciences). The sorted cells were washed with cold PBS twice, and the RNA was extracted using RNeasy Plus Mini Kit (Qiagen).

For detecting phosphorylated proteins in CD11b^+^Gr-1^+^ cells, cell treatment and flow cytometric analysis were modified according to the previous report (24). BM cells were deprived of serum and cytokines at least 30 min. Then, cells were stimulated with GlcN, collected at the indicated time points, and fixed immediately with 4% paraformaldehyde (Merck, Darmstadt, Germany). The ice-cold 100% methanol (Merck) was used for permeabilization. Cells were blocked with mice Fc Blocker for 15 min, and stained with directly conjugated CD11b, Gr-1, ERK1/2-pT202/pY204 (20A) and Stat3-pY705 (4/P-STAT3) antibodies (BD Biosciences) (Supplementary Table 5) for 30 min. Flow cytometry analyses were performed on a CytoFLEX S flow cytometer (Beckman Coulter, Brea, CA, USA) and results were analyzed with FlowJo software (TreeStar, Ashland, OR, USA).

### *In vitro* MDSC Differentiation

BM cells were resuspended in RPMI 1640 (Gibco) containing 10% FBS, 10 ng/mL interleukin (IL)-6, 10 ng/mL granulocyte macrophage colony-stimulating factor (GM-CSF), 10 ng/mL granulocyte (G)-CSF, 0.2 ng/mL transforming growth factor-β, and 50 μM 2-mercaptoethanol at 1 × 10^6^ cells/mL. Next, 0.5 mL resuspended cells were cultured in a 24-well plate. GlcN were added to the cells and incubated at 37°C and 5% CO_2_ for 72 h. The cells were washed and stained with direct conjugated antibodies.

### Colony Forming Analysis

The methylcellulose-based medium MethoCult™ M3434 was obtained from StemCell Technologies (Vancouver, Canada). Colony-forming assays were performed as previously described (22). To detect burst-forming unit-erythroids and other types of colonies, including oligopotential progenitor colony-forming unit (CFU)-granulocytes; erythrocytes, monocytes/macrophages, and megakaryocytes (GEMM); lineage-restricted progenitor CFU-granulocyte and monocytes/macrophages (GM); and precursors of granulocytes (G) and monocytes/macrophages (M), 2 × 10^4^ BM cells were plated in duplicate on MethoCult™ M3434 methylcellulose medium according to the manufacturer's protocol. Colonies were counted after 10 days of culture.

### Gene Expression Analysis

RNA isolation and first-strand cDNA synthesis were performed using the RNeasy Plus Mini Kit (Qiagen, Hilden, Germany) and RevertAid First-Strand cDNA Synthesis Kit (Thermo Fisher Scientific, Waltham, MA, USA) following the manufacturer's protocols. The indicated transcripts in cDNA samples were quantified using Fast SYBR™ Green Master Mix (Applied Biosystems, Foster City, CA, USA) according to the manufacturer's instructions. The reaction and signal detection were measured with a QuantStudio 3 Real-Time PCR System (Applied Biosystems). The primer sets used for gene detection are listed in Table 1. The gene expression level relative to that of GAPDH was calculated using the difference in threshold cycle method for reverse transcription-quantitative PCR analysis.

**Table 1:** The qPCR primer list.

**Genes**	**Forward primer**	**Reverse primer**
Arg-1	5′–GGAATCTGCATGGGCAACCTGTGT−3′	5′–AGGGTCTACGTCTCGCAAGCCA−3′
C/EBP-α	5′–GCAAAGCCAAGAAGTCGGTGGA−3′	5′–CCTTCTGTTGCGTCTCCACGTT−3′
C/EBP-β	5′–GGGTTGTTGATGTTTTTGGTTT−3′	5′–GAAACGGAAAAGGTTCTCAAAA−3′
c-Jun	5′–CCTTCTACGACGATGCCCTC−3′	5′–GGTTCAAGGTCATGCTCTGTTT−3′
COX2	5′–CAGACAACATAAACTGCGCCTT−3′	5′–GATACACCTCTCCACCAATGACC−3′
GAPDH	5′–CATGGCCTTCCGTGTTCCTA−3′	5′–GCGGCACGTCAGATCCA−3′
GATA-1	5′–CATTGGCCCCTTGTGAGGCCAGAGA−3′	5′–ACCTGATGGAGCTTGAAATAGAGGC−3′
G-CSF	5′–ATCCCGAAGGCTTCCCTGAGTG−3′	5′–AGGAGACCTTGGTAGAGGCAGA−3′
GM-CSF	5′–ACCAC CTATGCGGATTTCAT−3′	5′–TCATTACGCAGGCACAAAAG−3′
IL-6	5′–TCTGGGAAATCGTGGAAATGAG−3′	5′–TCTCTGAAGGACTCTGGCTTTGTC−3′
iNOS	5′–GAGACAGGGAAGTCTGAAGCAC−3′	5′–CCAGCAGTAGTTGCTCCTCTTC−3′
IRF8	5′–GATCGAACAGATCGACAGCA−3′	5′–GCTGGTTCAGC TTTGTCTCC−3′
PD-L1	5′–GTGAAACCCTGAGTCTTATCC−3′	5′–GACCATTCTGAGACAATTCC−3′
PU.1	5′–AGAAGCTGATGGCTTGGAGC−3′	5′–GCGAATCTTTTTCTTGCTGCC−3′
RB1	5′–GAACATCG AATCATGGAATCCCT−3′	5′–AGAGGACAAGCAGATTCAAGGTGAT−3′
Glut1	5′–CAGTTCGGCTATAACACTGGTG−3′	5′–GCCCCCGACAGAGAAGATG−3′
Glut2	5′–GTTGGAAGAGGAAGTCAGGGCA−3′	5′–ATCACGGAGACCTTCTGCTCAG−3′
Glut3	5′–CCGCTTCTCATCTCCATTGTCC−3′	5′–CCTGCTCCAATCGTGGCATAGA−3′
Glut4	5′–TACCTCCAGGTTGAAGGAACAGCAG−3′	5′–AGAGCCTGTGTGGCAAGAGTTCAGTG−3′

### *In vitro* MDSC Immunosuppressive Activity Assay

To analyze the immunosuppressive activity of MDSCs, the assay was performed according to the previous report (25). Murine CD11b^+^Gr1^+^ MDSCs were isolated from BM cells by using BD FACS Melody cell sorter. Murine splenocytes were incubated with 5 uM carboxylfluorescein succinimidyl ester (CFSE) (Biolegend, San Diego, CA, USA) for 5 min, and then washed with RPMI 1640 twice. The sorted CD11b^+^Gr1^+^ MDSCs were immediately co-cultured with CFSE-stained splenocytes at a density of 5 × 10^5^ cells per well at a ratio of 1:4 (MDSCs:T cells) in the presence of anti-CD3 (145-2C11) and anti-CD28 (37.51) antibodies, which were purchased from BD Biosciences and used for T cell activation. After 48 h, T-cell proliferation was evaluated by CFSE dilution by flow cytometry. The suppressive ability of MDSCs was expressed as percentage of CD8^+^ T cell proliferation (Supplementary Table 6).

### Western Analysis

To examine the influence of GlcN in ERK1/2 and STAT3 activation, the CD11b^+^ cells were isolated from BM cells by using anti-APC Magnetic Particles (see above). The sorted CD11b^+^ cells were incubated at 37°C in IMDM medium supplemented with 1% BSA for at least 30 min. Then, sorted CD11b^+^ cells (1.5 × 10^6^ cells/mL) were treated with 1 mM GlcN for 0, 5, 15, and 30 min. Cells were collected, and lysed with RIPA buffer (50 mM Tris-HCl, pH 7.4; 150 mM NaCl; 1% Triton X-100; 0.1% SDS; 1 mM EDTA; 1 mM phenylmethylsulfonyl fluoride; 10 μg/ml aprotinin; 10 μg/ml leupeptin; 10 μg/ml pepstatin; 1% sodium deoxycholate; 1 mM sodium fluoride; 1 mM sodium orthovanadate; 25 mM β-glycerophosphate). All reagents used for RIPA buffer preparation were purchased from Sigma-Aldrich. Western blot was performed with the following antibodies: Anti-phospho-STAT3 antibody (Tyr705) (M9C6), anti-STAT3 antibody (79D7), anti-phospho-p44/42 MAPK (Thr202/Tyr204) antibody (polyclonal) and anti-p44/42 MAPK antibody (polyclonal). All of these antibodies were purchased from Cell Signaling Technology. Detections were performed using Clarity Western ECL Substrate (Bio-Rad, USA), and digital images were captured by using Amersham Imager 680 (GE Healthcare Lifescience, Chicago, IL, USA). For detection of phosphorylated form and total proteins on the same membrane, the phosphorylated proteins were firstly detected, and then the membrane was stripped by using stripping reagent (Bionovas, Toronto, Ontario, Canada) before re-probing with another antibody.

### Statistical Analysis

Statistical analyses were performed using GraphPad Prism 6 software (GraphPad, Inc., La Jolla, CA, USA). For two groups, Student's *t* test was used; for multiple groups, one-way analysis of variance followed by Tukey multiple comparison test was used. Values are presented as the mean ± standard deviation. *p* < 0.05 was considered to indicate statistically significant results.

## Results

### GlcN Supplement Promotes MDSC Production

**Figure 1:**
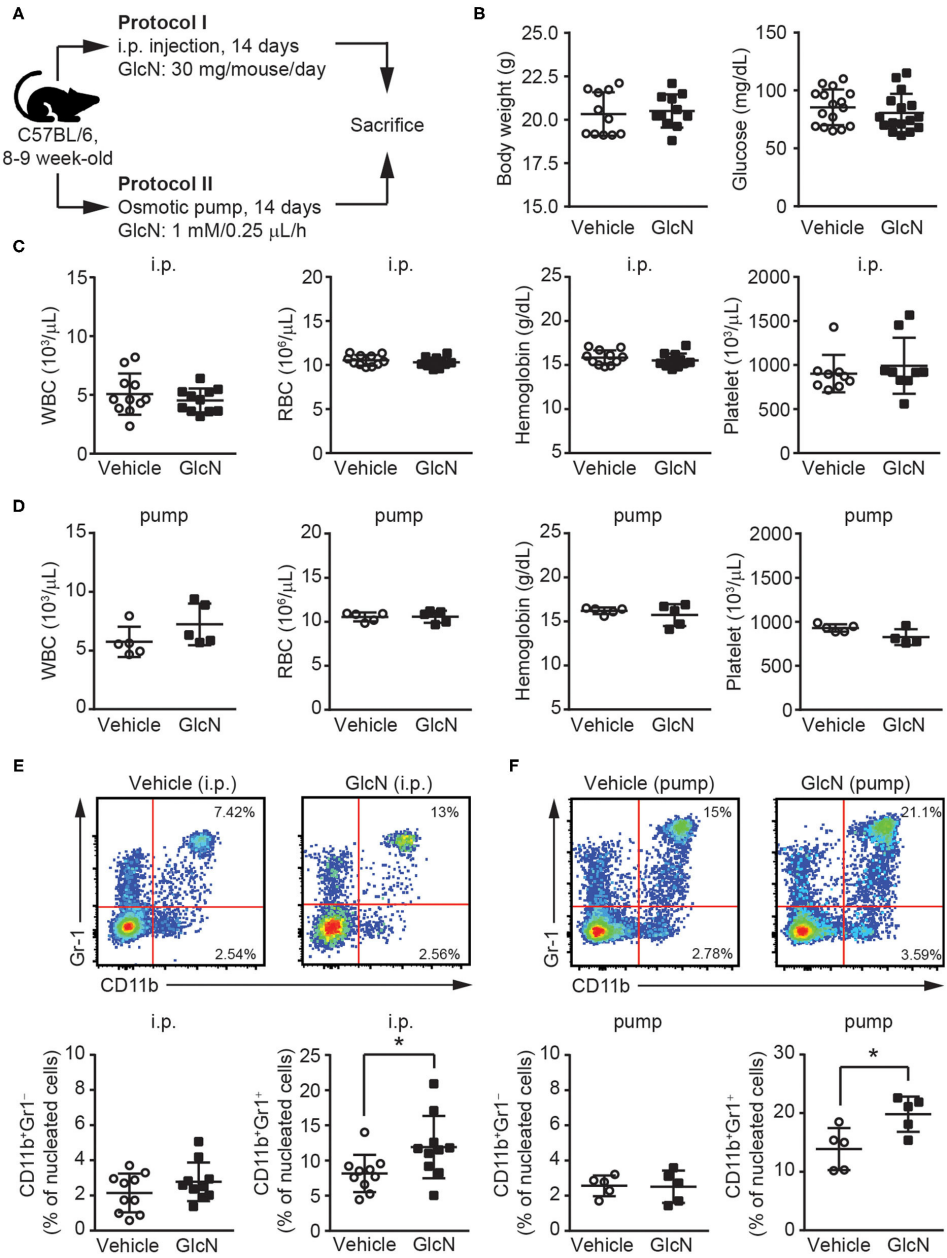
GlcN expands the myeloid compartment of mice. **(A)** Schematic of GlcN supplementation methods to mice. **(B)** Body weight and fasting glucose level of GlcN-supplemented mice after 14-day intraperitoneal (i.p.) injection. **(C)** WBC count, RBC count, hemoglobin level, and platelet count following i.p. treatment. **(D)** WBC count, RBC count, hemoglobin level, and platelet count following 14-day pump release. **(E)** Myeloid compartment of peripheral blood from mice given i.p. injection. **(F)** Myeloid compartment of peripheral blood from osmotic pump-treated mice. The results are presented as the mean ± standard deviation ^*^P < 0.05.

To determine the effect of GlcN on hematopoietic reprogramming, GlcN was delivered into C57BL/6 mice using two different methods, i.p. injection according to a previous report (20) and osmotic pump release for maintaining a steady GlcN concentration (Figure 1A). Supplementation with GlcN did not change the body weight or fasting glucose level of the mice after i.p. injection (Figure 1B). The white blood cell (WBC) count, red blood cell (RBC) count, hemoglobin level, and platelet count of the mice were not affected by treatment with GlcN by i.p. injection (Figure 1C) and pump release (Figure 1D). Notably, the frequency of CD11b^+^Gr1^+^ MDSCs in the peripheral blood (PB) were elevated after GlcN supplementation via i.p. injection, whereas that of CD11b^+^Gr1^−^ myeloid cells were not (Figure 1E). Similar results were observed in the pump release group (Figure 1F).

**Figure 2:**
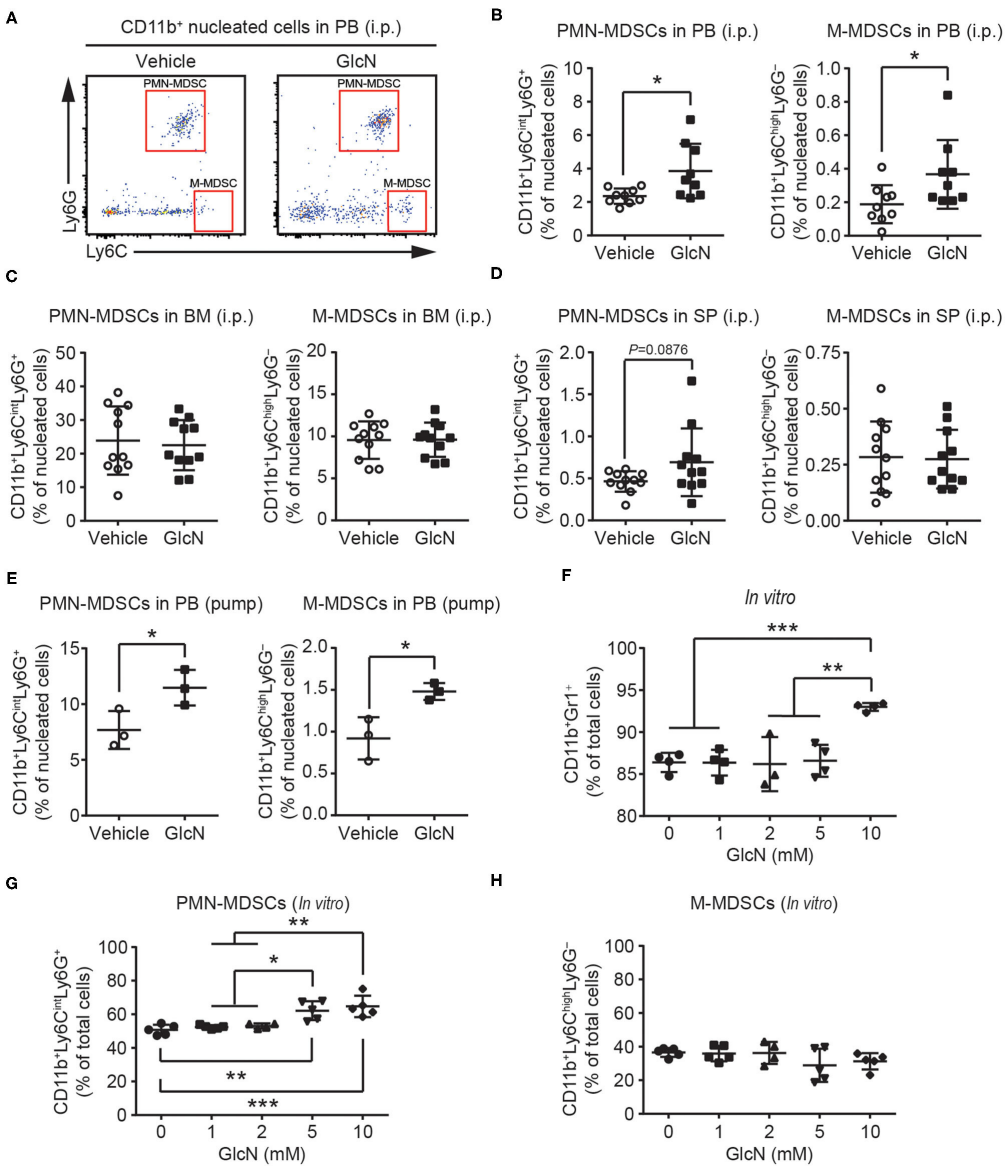
GlcN supplementation expands the compartment of MDSCs. **(A)** Schematic of PMN-MDSC and M-MDSC flow cytometry gating. PMN-MDSC and M-MDSC levels in **(B)** peripheral blood (PB), **(C)** bone marrow (BM), **(D)** spleen (SP) following i.p. injection. **(E)** PMN-MDSC and M-MDSC levels in the PB following pump release. Wild-type BM cells were stimulated with IL-6, GM-CSF, G-CSF, and transforming growth factor-β (TGF-β) to induce MDSC expansion *in vitro*. **(F)** Expanded CD11b^+^Gr1^+^ MDSC frequency. **(G)** Expanded PMN-MDSC frequency. **(H)** Expanded M-MDSC frequency. The quantified results are presented as the mean ± standard deviation. *P < 0.05; **P < 0.01; ***P < 0.001.

Next, polymorphonuclear and monocytic subpopulations of MDSCs (defined as PMN- and M-MDSCs, respectively) in the CD11b^+^ myeloid cell compartments were examined by flow cytometry (Figure 2A). Similar to the results for CD11b^+^Gr1^+^ MDSCs (Figures 1E,F), both PMN- and M-MDSCs were elevated in the PB of mice following i.p. injection of GlcN (Figure 2B). Consistent with the results for the i.p. injection method, GlcN delivery via pump release elevated PMN- and M-MDSCs in the PB (Figure 2E). However, 14-day i.p. GlcN treatment did not further increase PMN- and M-MDSCs in BM (Figure 2C) and SP (Figure 2D). The *in vitro* myeloid cell differentiation assay using murine BM cells supported that GlcN enhanced the production of CD11b^+^Gr1^+^ MDSCs (Figure 2F). Moreover, GlcN supplementation favored the production of PMN-MDSCs (Figure 2G) rather than of M-MDSCs *in vitro* in our differentiation model (Figure 2H). These results suggest that GlcN treatment interferes with myelopoiesis to promote MDSC production.

### GlcN Affects the Function of Hematopoietic Stem and Progenitor Cells

**Figure 3:**
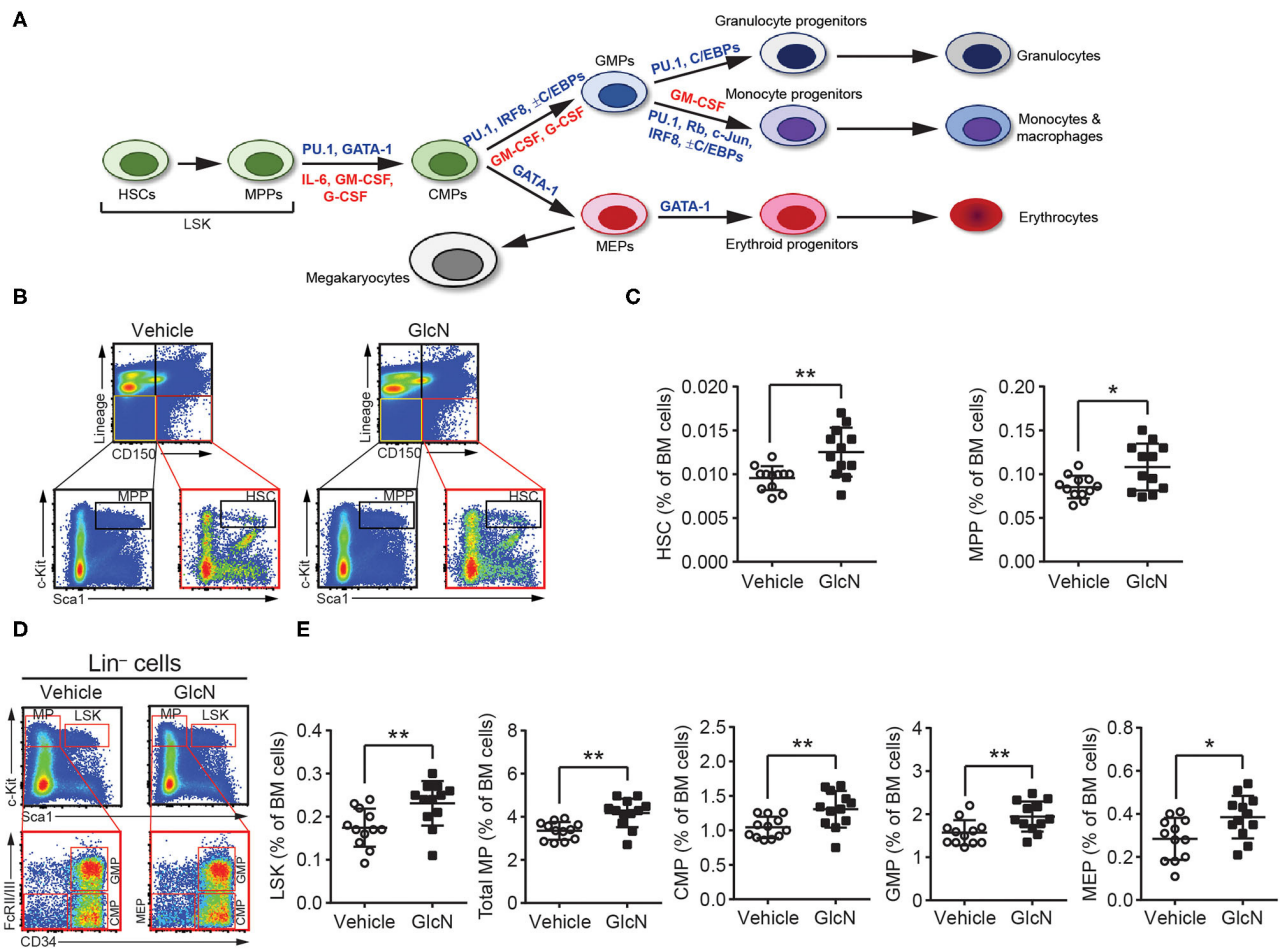
GlcN increases hematopoietic stem and progenitor cell compartment. **(A)** Schematic of hematopoietic hierarchy and critical transcriptional factors and cytokines in myelopoiesis. **(B)** Schematics of identifying hematopoietic stem cells (HSCs) and multipotent progenitor cells (MPPs). **(C)** The frequency of HSCs and MPPs in BM after i.p. injection. **(D)** Schematics of identifying Lin^−^c-Kit^+^Sca-1^+^ cells (LSKs), total myeloid progenitors (MPs, Lin^−^c-Kit^+^Sca-1^−^), common myeloid progenitors (CMPs, Lin^−^c-Kit^+^Sca-1^−^CD34^+^FcRII/III^−^), granulocyte-macrophage progenitors (GMPs, Lin^−^c-Kit^+^Sca-1^−^CD34^+^FcRII/III^+^) and megakaryocyte-erythroid progenitors (MEP, Lin^−^c-Kit^+^Sca-1^−^CD34^−^FcRII/III^−^). **(E)** The frequency of LSKs, MPs, CMPs, GMPs and MEPs in BM after i.p. injection. The quantified results are presented as the mean ± standard deviation. *P < 0.05; **P < 0.01.

As MDSCs are a heterogeneous population of immature myeloid cells, we focused on whether GlcN supplementation affected HSPC compartment and myelopoiesis-related gene expression (Figure 3A). To determine the impact of GlcN supplementation on HSPCs, we examined different subpopulations of HSPCs isolated from the BM of injected mice. GlcN significantly elevated the population of hematopoietic stems cells (HSCs), which locate at the top of the hematopoietic hierarchy, as well as multipotent progenitor cells (MPPs) in BM (Figure 3C). The representative flow cytometric figures and gating strategies for HSC and MPP analysis were shown in Figure 3B. HSCs and MPPs are contained in the Lin^−^c-Kit^+^Sca-1^+^ (LSK) cell population, which expresses Sca-1 and c-Kit but lacks the lineage (Lin) markers expressed on mature myeloid and lymphoid cells. LSK cells and the following myeloid progenitor cells: total myeloid progenitors (MPs, Lin^−^c-Kit^+^Sca-1^−^), common myeloid progenitors (CMPs, Lin^−^c-Kit^+^Sca-1^−^CD34^+^FcRII/III^−^), granulocyte-macrophage progenitors (GMPs, Lin^−^c-Kit^+^Sca-1^−^CD34^+^FcRII/III^+^), and megakaryocyte-erythroid progenitors (MEP, Lin^−^c-Kit^+^Sca-1^−^CD34^−^FcRII/III^−^) in the BM were analyzed by flow cytometry (Figure 3D). Consistent with the results of HSCs and MPPs, GlcN significantly elevated the population of various types of hematopoietic progenitor cells (Figure 3E).

**Figure 4:**
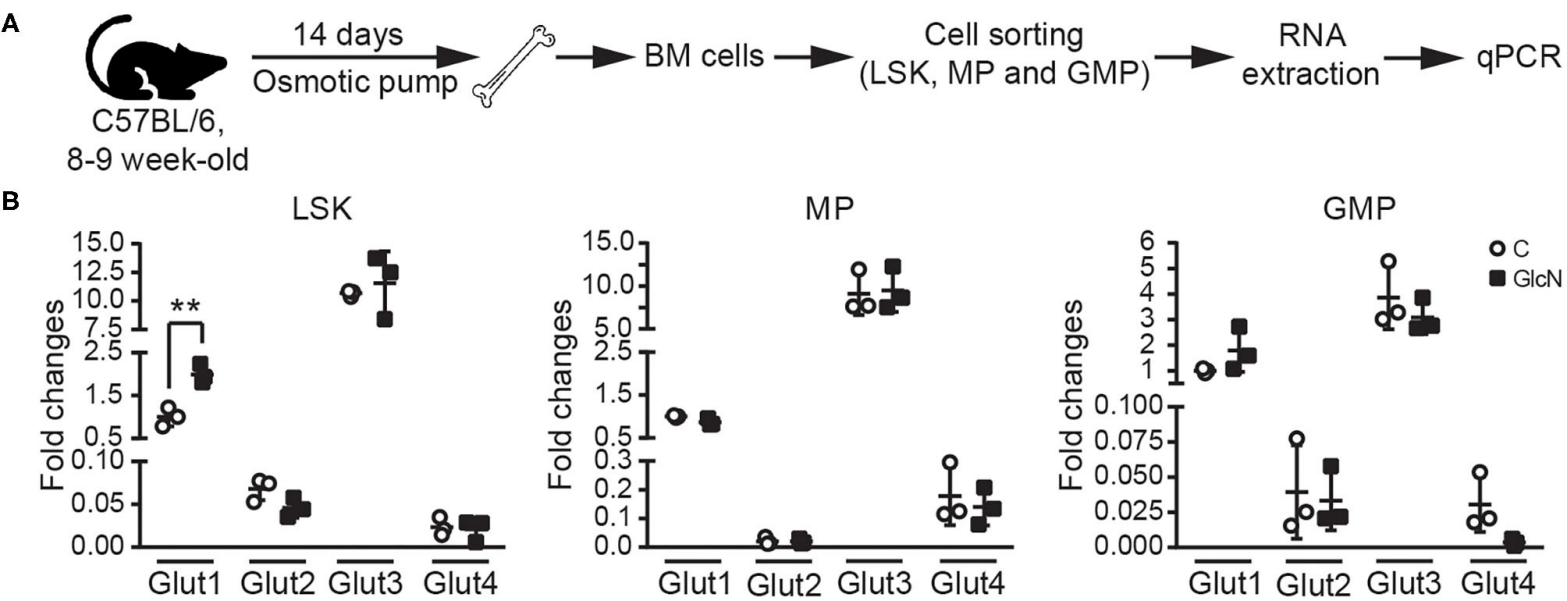
The influence of GlcN treatment in the expression of glucose transporters of hematopoietic stem and progenitor cells. **(A)** The schematic of glucose transporter expression analysis in sorted LSKs, MPs, and GMPs. **(B)** Relative mRNA expression of Glut1, 2, 3 and 4 in LSKs, MPs, and GMPs were determined by quantitative reverse transcription-PCR. GlcN treatment significantly increase Glut1 expression in LSKs. The quantified results are presented as the mean ± standard deviation. ^**^P < 0.01.

**Figure 5:**
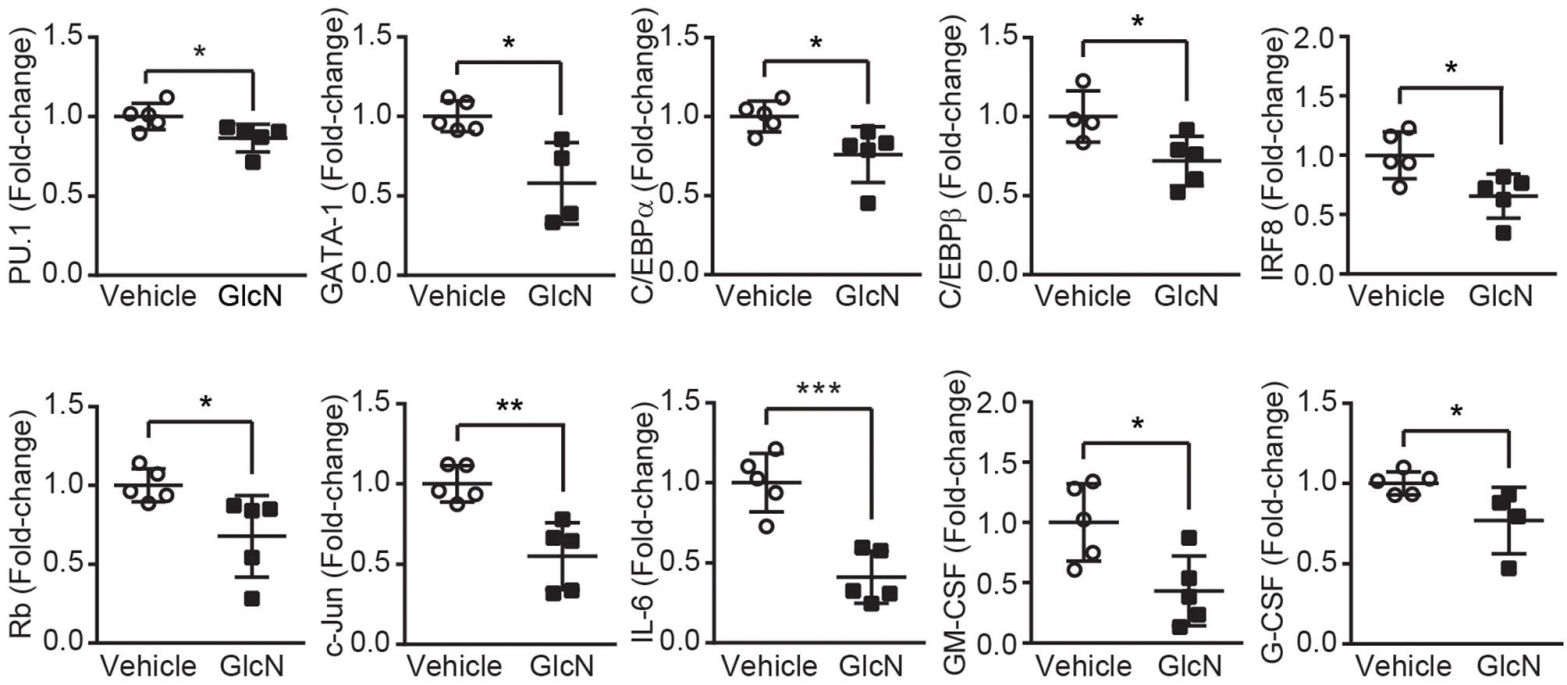
GlcN suppresses the expression of transcriptional factors and cytokines involved in myelopoiesis. Relative mRNA expression of PU.1, GATA1, C/EBPα, C/EBPβ, IRF8, Rb, c-Jun, IL-6, GM-CSF, and G-CSF in the BM cells of GlcN-treated mice were determined by quantitative reverse transcription-PCR. The quantified results are presented as the mean ± standard deviation. *P < 0.05; **P < 0.01; ***P < 0.001.

Previous studies indicated glucose transporters facilitate GlcN transport (3, 26). In hematopoietic system, differently expressed Glut1 and 4 affect glucose uptake during erythropoiesis (27), and disruption of Glut1 in HSCs interferes myelopoiesis (28). Thus, LSKs, MPs and GMPs in BM were isolated from the pump release groups, and the expression of Glut1, 2, 3, and 4 were analyzed (Figure 4A). Our results indicated that GlcN treatment significantly increase Glut1 expression in LSKs (Figure 4B). Because GlcN treatment expanded MDSCs in the PB, the critical transcription factors and cytokines involved in myelopoiesis (Figure 3A) were analyzed. GlcN treatment significantly suppressed the expression of these transcription factors, including PU.1, GATA1, C/EBPα, C/EBPβ, IRF8, Rb, c-Jun, as well as cytokines, including IL-6, GM-CSF, and G-CSF in BM cells (Figure 5). These results suggest that HSPCs might uptake GlcN through Glut1, and then GlcN weakens the differentiation function of HSPCs.

**Figure 6:**
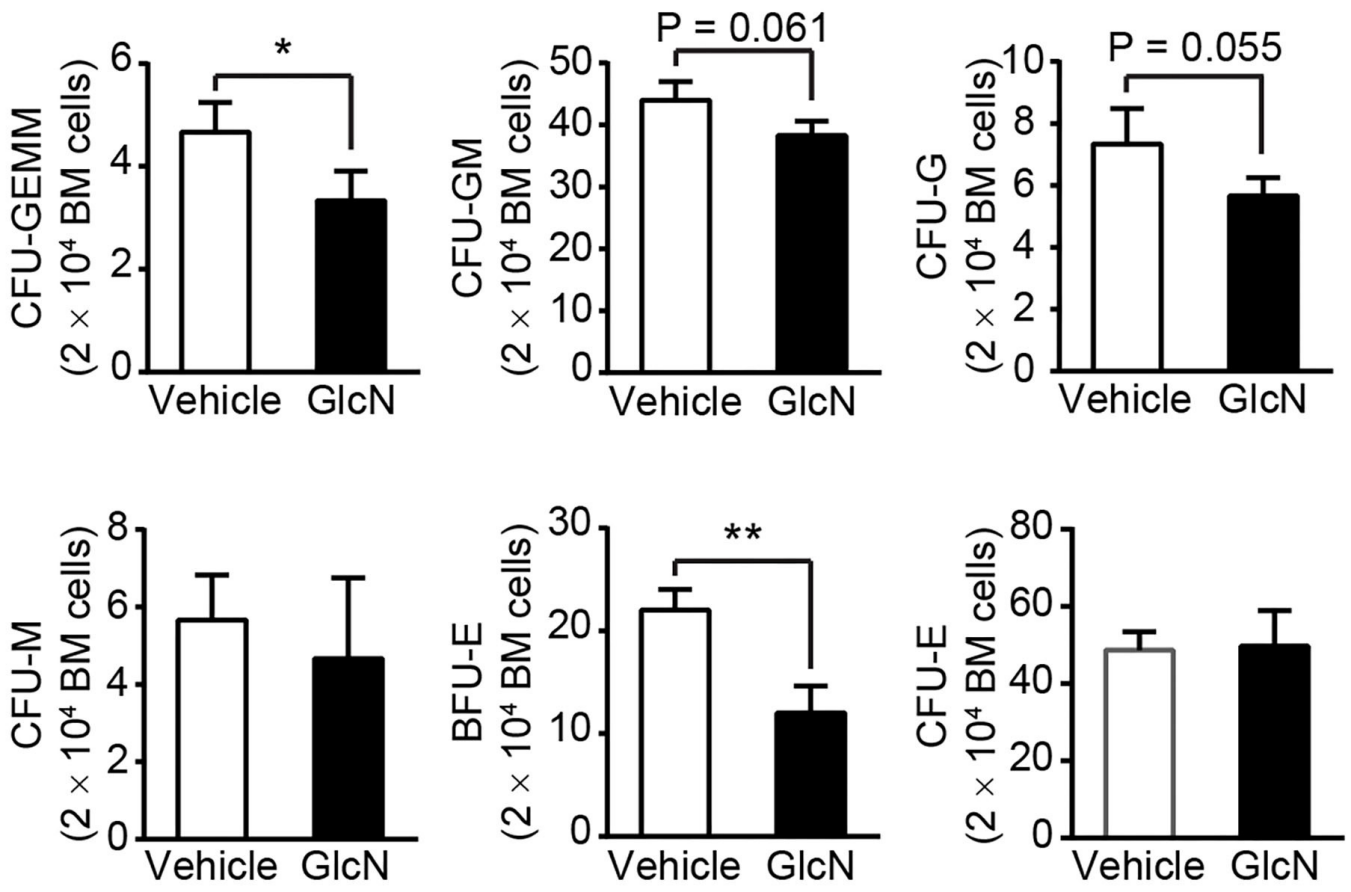
GlcN treatment weakens hematopoietic stem and progenitor cell differentiation. BM cells from i.p.-injected mice were extracted and directly cultured in methylcellulose-based medium M3434. Colonies were counted after 10 days of culture. Quantification of CFU-GEMM, CFU-GM, CFU-G, CFU-M, BFU-E and CFU-E. Quantified results are presented as the mean ± standard deviation. *P < 0.05; **P < 0.01.

To further examine whether GlcN affected the proliferation and differentiation capacity of HSPCs, BM cells isolated from control or GlcN-treated mice were subjected to colony-forming unit (CFU) analysis. Consistent with the transcription factor expression, the ability of HSPCs to form CFU-granulocytes, erythrocytes, monocytes/macrophages, and megakaryocytes (GEMM); CFU-granulocytes and monocytes/macrophages (GM); and CFU-granulocytes (G) were weakened. However, the CFU-monocytes/macrophages (M) were not affected. Interestingly, the earlier erythroid progenitors, burst-forming unit erythroid (BFU-E) cells, were decreased, whereas more mature erythroid progenitor cells, named as CFU-erythrocytes (E), were not affected in GlcN-supplemented mice (Figure 6).

### GlcN Supplement Enhances the Immunosuppressive Ability of MDSCs

**Figure 7:**
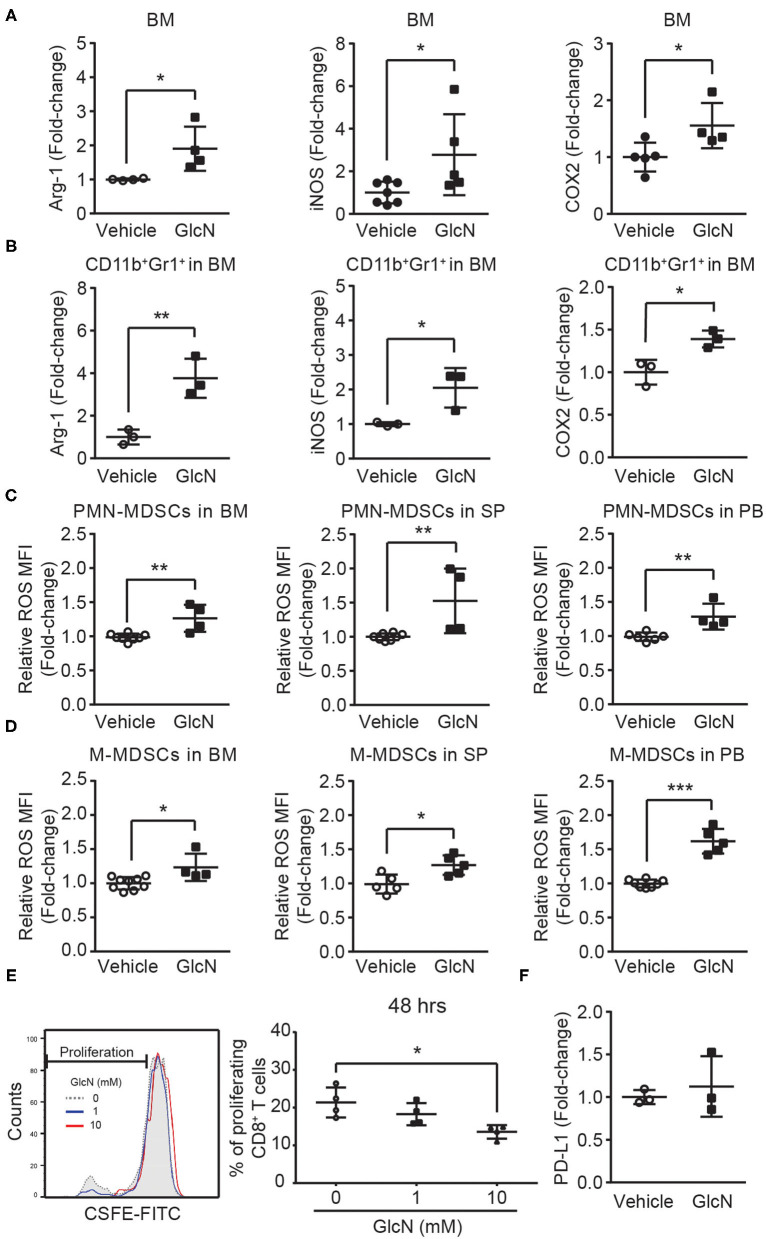
GlcN treatment enhances the immunosuppressive ability of MDSCs. Mice were treated with GlcN by i.p. injection for 14 days. Arg-1, iNOS, and COX2 expression levels in **(A)** total BM cells and **(B)** BM CD11b^+^Gr1^+^ MDSCs. ROS levels in **(C)** PMN-MDSCs and **(D)** M-MDSCs isolated from BM, SP, and PB. **(E)** T cell immunosuppressive ability of BM CD11b^+^Gr1^+^ MDSCs. **(F)** Quantification of PD-L1 expression levels in BM cells. MFI: Mean fluorescence intensity. Quantified results are presented as the mean ± standard deviation. *P < 0.05; **P < 0.01; ***P < 0.001.

To study the impact of GlcN on MDSC functions, genes associated with MDSC functions were examined. Cyclooxygenase 2 (COX2) is an important upstream regulator of Arg-1 and iNOS in MDSCs (29). *In vivo* GlcN treatment increased the expression of Arg-1, iNOS, and COX2 in BM cells (Figure 7A), as well as in CD11b^+^Gr1^+^ MDSCs isolated from the BM (Figure 7B). Since the increase of Arg-1 might contribute to ROS accumulation in cells, ROS levels in PMN- and M-MDSCs, which were isolated from BM, SP and PB in GlcN treated mice, were analyzed. *In vivo* GlcN treatment elevated ROS levels in both PMN-MDSC and M-MDSCs (Figures 7C,D).

To further examine the impact of GlcN on the immunosuppressive ability of MDSCs, MDSC-mediated suppression in CD8^+^ T cell proliferation was assessed. After 48 h of co-culture of CD11b^+^Gr1^+^ MDSCs and splenocytes, GlcN significantly enhanced the suppressive ability of MDSCs in a dose-dependent manner (Figure 7E). Previous results indicated that the expression of programmed death-ligand 1 (PD-L1) on MDSCs is involved in MDSC-mediated T-cell suppression (30). However, GlcN treatment did not influence PD-L1 expression (Figure 7F). These results indicate that GlcN enhanced the immunosuppressive ability of MDSCs through ROS production, but did not affect PD-L1 expression.

### GlcN Activates the STAT3 and ERK1/2 Signaling Pathway in MDSCs to Modulate Immunosuppressive Ability

**Figure 8:**
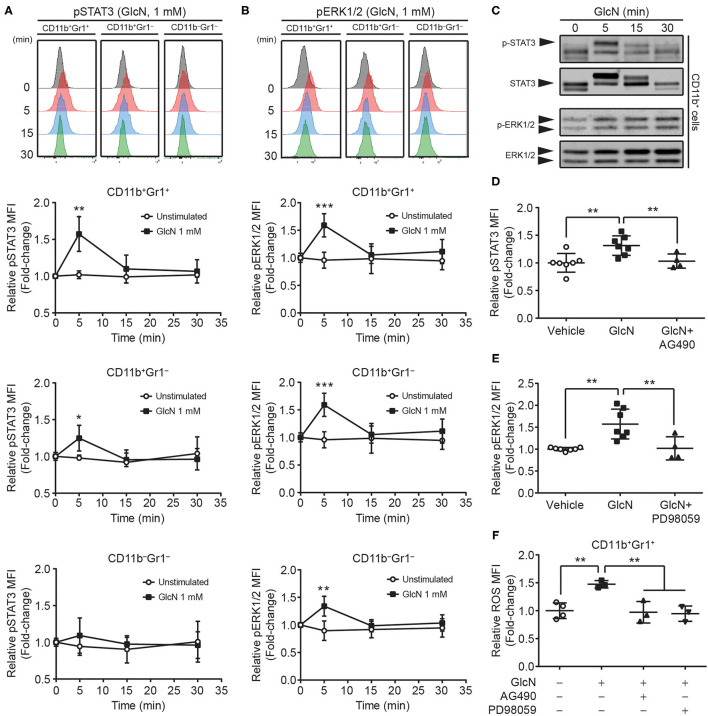
GlcN stimulates the activation of STAT3 and ERK1/2 in MDSCs. The level and mean fluorescence intensity (MFI) of **(A)** phosphorylated STAT3 (pSTAT3) and **(B)** phosphorylated ERK1/2 (pERK1/2) in BM CD11b^+^Gr1^+^, CD11b^+^Gr1^−^, and CD11b^−^Gr1^−^ cells were shown. **(C)** The levels of phospho-STAT3, STAT3, phospho-ERK1/2 and ERK1/2 in GlcN-treated CD11b^+^ cells were detected by Western analysis. **(D)** JAK2 inhibitor AG490 and **(E)** MEK inhibitor PD98059 were used to suppress GlcN-induced phosphorylation of STAT3 and ERK1/2 proteins, respectively, in BM CD11b^+^Gr1^+^ cells. **(F)** Inhibition of either STAT3 or ERK1/2 decreased GlcN-promoted ROS production in CD11b^+^Gr1^+^ MDSCs. Quantified results are presented as the mean ± standard deviation. *P < 0.05; **P < 0.01; ***P < 0.001.

STAT3 and ERK1/2 signaling pathways are involved in the MDSC-mediated suppressive function (31, 32). To determine whether GlcN treatment influenced these two signaling pathways to modulate MDSC functions, the phosphorylation of STAT3 and ERK1/2 in MDSCs was analyzed. BM cells were stimulated with GlcN (1 mM) for 5, 10, and 30 min, and the phosphorylation of STAT3 (Figure 8A) and ERK1/2 (Figure 8B) was examined by flow cytometry. Phosphorylation of STAT3 reached a peak after 5 min of GlcN stimulation in CD11b^+^Gr1^+^ MDSCs and CD11b^+^Gr1^−^ myeloid cells, but not in non-myeloid cells (CD11b^−^Gr1^−^ cells) (Figure 8A). GlcN stimulation also increased the phosphorylation of ERK1/2 in CD11b^+^Gr1^+^ MDSCs, CD11b^+^Gr1^−^ myeloid cells, and CD11b^−^Gr1^−^ non-myeloid cells (Figure 8B). Furthermore, Western analysis was used to confirmed the results from flow cytometry. The GlcN treatment increased the phosphorylation of STAT3 and ERK1/2 in sorted CD11b^+^ cells (Figure 8C).

The AG490 (JAK2 inhibitor) and PD98059 (MEK inhibitor) have been extensively used to inhibit the JAK2–STAT3 and MEK–ERK1/2 pathways, respectively. Inhibition of these pathways reversed GlcN-mediated activation of STAT3 and ERK1/2, respectively (Figures 8D,E). Furthermore, inhibition of either the JAK2/STAT3 or MEK/ERK1/2 pathway downregulated GlcN-promoted ROS production in CD11b^+^Gr1^+^ MDSCs (Figure 8F). Our results clearly demonstrated that GlcN activates the STAT3 and ERK1/2 pathway to enhance the immunosuppressive ability of MDSCs.

## Discussion

**Figure 9:**
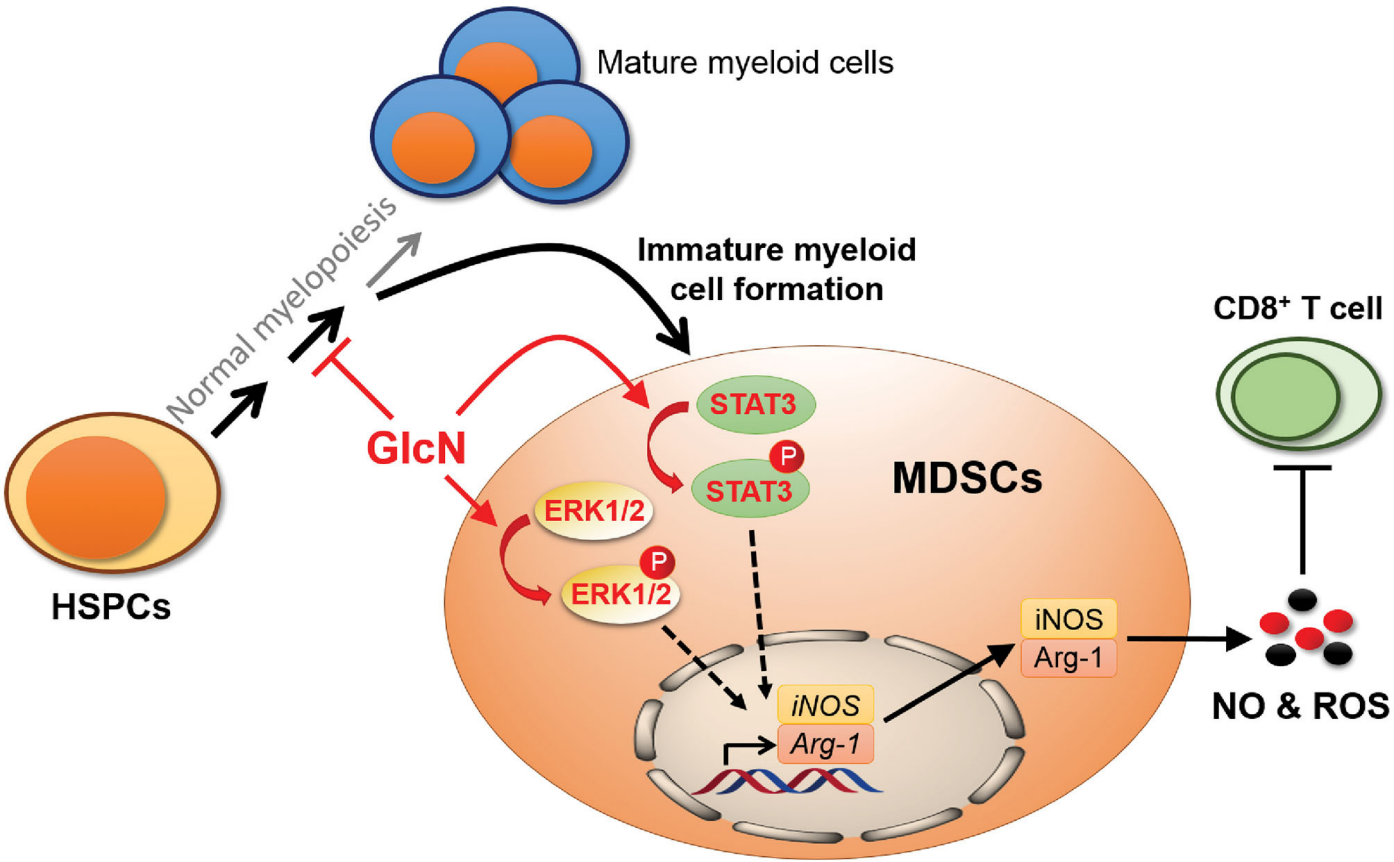
Schematic illustration showing the influence of GlcN in myelopoiesis and MDSC functions. GlcN interferes myelopoiesis by downregulating the gene expression of critical transcription factors and cytokines involved in HSPC differentiation and myelopoiesis to produce more immature myeloid cells, MDSCs. In addition, GlcN increases STAT3 and ERK1/2 phosphorylation to enhance the MDSC immunosuppressive ability.

Although previous studies demonstrated that GlcN has beneficial effects against joint pain, cancers, and anti-inflammation, the influence of GlcN on hematopoiesis and immune cell function is largely unclear. Here, we demonstrated that GlcN treatment influences hematopoiesis to increase the production of MDSCs *in vivo*. This occurs because GlcN interferes with HSPC function and myelopoiesis by downregulating the gene expression of key transcription factors and cytokines involved in HSPC differentiation and myelopoiesis. We also demonstrated that GlcN directly activates the STAT3 and ERK1/2 pathways to enhance the immunosuppressive function of MDSCs (Figure 9).

Previous reports indicated that the CXC chemokine receptor 2 on GMPs contributes to M-MDSC generation via ERK1/2 and STAT3 pathways (33); activated STAT3 regulates Arg-1 and iNOS expression to enhance MDSC activities (34), whereas inhibition of STAT3 downregulates IRF8 expression to suppress myeloid cell differentiation (35). Our results revealed a novel function for GlcN in the regulation of MDSC function through the STAT3 and ERK1/2 pathways. While, we could not rule out the possibility that GlcN might activate ERK1/2 and STAT3 pathways to enhance MDSC production in mice. Similar to our results about GlcN-mediated enhancement of MDSC immunosuppressive activity on cytotoxic T cells via ERK1/2 pathway, GlcN prolongs ERK1/2 phosphorylation to suppress the cytotoxic activity of NK-92 natural killer (NK) cells (36). Accumulating data has highlighted the interaction between immune cells, such as MDSCs and NK cells, and cancer cells within the tumor microenvironment to promote cancer progression (37). Numerous results indicated that increasing MDSC infiltration and low NK activity in the tumor microenvironment contribute to immunosuppression (37, 38). Therefore, the concern whether GlcN influences immune cell activities in tumor microenvironment was arose.

However, the study in somatic cancer cell lines indicated that GlcN elicits the anti-cancer effect via the downregulation of STAT3 and ERK1/2 pathways. GlcN decreased STAT3 activation to reduce the expression of anti-apoptosis protein, surviving, in prostate carcinoma DU145 cells (39); impaired the stemness of human aldehyde dehydrogenase-positive breast cancer stem cells (40). GlcN also modulated cell proliferation via the decreasing of ERK1/2 phosphorylation in A549 lung cancer cells (40). The controversy about distinct impacts of GlcN in immune cells and cancer cells has to be further investigated in the proper mouse model to evaluate the *in vivo* effect of GlcN on the interplay between immune cells and cancer cells, which localize in the same tumor microenvironment.

Our results and the discussion above rose a potential immune risk for people who intake glucosamine. The inflammatory response is a defense mechanism that protects us from infection and injury. The fine-tuning of inflammation is important to create a favorable environment for the resolution phase to take place and for the body homeostasis to return. However, uncontrolled or unresolved inflammation can lead to tissue damage or give rise to chronic inflammatory diseases, including cancers, autoimmune diseases and metabolic syndromes (41). According to a detail review about biosafety, side effects and glucose metabolism of GlcN in animals and human, the LD50 for GlcN for animals exceeds 5,000 mg/kg, and there are no adverse effects in both animals and human from long-term studies. Oral administration of large doses of GlcN in animals has no documented effects on glucose metabolism, however. fasting plasma glucose values are decreased slightly for human after 66-week oral GlcN uptake (26). In our study, we revealed the novel *iv vivo* functions of GlcN in myelopoiesis and MDSC functions. MDSCs have been most intensively studied in the context of cancers, and the main biological function attributed to MDSC is the immunosuppression of T cells. Recent reports revealed a predominant expansion of circulating MDSC subsets in cancer patients exceeds the expansion in infectious and inflammatory diseases, while the low MDSC frequency presents in healthy individuals (42, 43). MDSCs are a very heterogeneous group of immature myeloid cells. The phenotypic, morphological and functional heterogeneity of these cells generates a lot of confusion in investigation and analysis of their roles in inflammatory responses. Some results revealed morphologic, molecular, and functional differences in tumor-induced MDSC subpopulations. The phenotype and cellular composition of M-MDSCs changes during tumor growth, while that of PMN-MDSCs remains static. M-MDSCs and PMN-MDSCs suppress T-cell responses through different signaling pathways in mouse T-cell lymphoma model (44). Until now, numerous studies document that MDSCs strongly influence cancer outcomes, but are less informative regarding the relevance of MDSC with infection, autoimmunity, transplantation and aging, especially in humans (45). The influence of GlcN in immune system encounters the similar challenge. The details of immune cell profiling and cytokine productions in healthy individuals and patients undergoing different kinds of diseases who intake glucosamine as dietary supplement are still unclear. Thus, more efforts are needed to develop standard protocols, proper animal models or comparable information platform to analyze, compare and investigate the biological functions of GlcN and MDSCs *in vivo* and in clinics.

Immune cell therapy is the novel clinical approach for treating incurable diseases. Currently, immune cell therapies have been developed to treat cancer, infectious diseases, and autoimmune disorders (46). MDSCs show potential for clinical application in transplantation and autoimmune diseases (47, 48). In patients who have undergone organ transplantation, MDSCs were shown to participate in establishing graft tolerance (49). Additionally, adoptive transfer of MDSCs into a collagen-induced rheumatoid arthritis mouse model reduced disease severity (50). However, the use of MDSCs in clinical treatment remains challenging, as it is difficult to harvest enough healthy autologous cells from patients who are ill for MDSC production (51). Combined with our findings, using GlcN to enhance the *in vitro* or *in vivo* generation of MDSCs may be beneficial for establishing an MDSC therapeutic method.

## Data Availability Statement

The raw data supporting the conclusions of this article will be made available by the authors, without undue reservation.

## Ethics Statement

The animal study was reviewed and approved by the Institutional Animal Care and Use Committee (IACUC) of National Yang Ming Chiao Tung University (Taipei Yang Ming campus) (IACUC number: 1081005).

## Author Contributions

EL, S-WC, and Y-IC designed the study and wrote the manuscript. EL performed most of the experiments. S-WC and L-KC implanted osmotic pumps and analyzed data. T-AL, Y-XW, and C-CJ discussed and made suggestions for this study. All authors contributed to the article and approved the submitted version.

## Funding

This work was supported by grants from the Ministry of Science and Technology (grants MOST 107-2320-B-010-022, 108-2320-B-010-005 and 109-2320-B-010-048) (Y-IC) and Yen Tjing Ling Medical Foundation (CI-109-6) (Y-IC). This work was also supported by the Yin Yen-Liang Foundation Development and Construction Plan of the College of Medicine, National Yang Ming Chiao Tung University (Y-IC).

## Conflict of Interest

The authors declare that the research was conducted in the absence of any commercial or financial relationships that could be construed as a potential conflict of interest.

## Publisher's Note

All claims expressed in this article are solely those of the authors and do not necessarily represent those of their affiliated organizations, or those of the publisher, the editors and the reviewers. Any product that may be evaluated in this article, or claim that may be made by its manufacturer, is not guaranteed or endorsed by the publisher.
