# Supplementary material for: Glucosamine Interferes With Myelopoiesis and Enhances the Immunosuppressive Activity of Myeloid-Derived Suppressor Cells
Source: Front Nutr. 2021 Nov 10;8:762363. doi: 10.3389/fnut.2021.762363 (PMC8660085; doi:10.3389/fnut.2021.762363)
Supplement: Supplementary file 1 [file Table_1.pdf]

Supplementary Table 1. The antibodies used for characterization of mouse hematopoietic stem cells

Specificity	Fluorochrome	Clone	Company	Expression[a]
CD41	FITC	MWReg30	BD Bioscience	−
CD48	FITC	HM48-1	BD Bioscience	−
Gr-1	FITC	RB6-8C5	BD Bioscience	−
Ter119	FITC	TER-119	BD Bioscience	−
B220	FITC	RA3-6B2	BD Bioscience	−
CD150	PE	Q38-480	BD Bioscience	−/+[a]
c-Kit	APC	2B8	BD Bioscience	+
Sca-1	BB700	D7	BD Bioscience	+

[a]$CD150^-$ expression refers to MPP, while $CD150^+$ expression refers to HSC.
