# Supplementary material for: Glucosamine Interferes With Myelopoiesis and Enhances the Immunosuppressive Activity of Myeloid-Derived Suppressor Cells
Source: Front Nutr. 2021 Nov 10;8:762363. doi: 10.3389/fnut.2021.762363 (PMC8660085; doi:10.3389/fnut.2021.762363)
Supplement: Supplementary file 2 [file Table_2.pdf]

Supplementary Table 2. The antibodies used for characterization of mouse myeloid progenitor cells

Specificity	Fluorochrome	Clone	Company	Expression [a]
CD3	Biotin	145-2C11	BD Bioscience	－
CD4	Biotin	GK1.5	BD Bioscience	－
CD8	Biotin	53-6.7	BD Bioscience	－
B220	Biotin	RA3-6B2	BD Bioscience	－
Ter119	Biotin	TER-119	BD Bioscience	－
Gr-1	Biotin	RB6-8C5	BD Bioscience	－
IgM	Biotin	R6-60.2	BD Bioscience	－
CD19	Biotin	1D3	eBioscience	－
IL-7Ra	Biotin	B12-1	BD Bioscience	－
CD34	FITC	RAM34	BD Bioscience	－/＋ [a]
FcγRIII/FcγRII	PE	2.4G2	BD Bioscience	－/＋ [a]
c-Kit	APC	2B8	BD Bioscience	＋
Streptavidin	PE-Cy™7	–	BD Bioscience	－
Sca-1	BB700	D7	BD Bioscience	＋

[a] $CD34^-$ and $Fc\gamma RIII/Fc\gamma RII^-$ expression refers to MEP; $CD34^+$ and $Fc\gamma RIII/Fc\gamma RII^-$ expression refers to CMP; $CD34^+$ and $Fc\gamma RIII/Fc\gamma RII^+$ expression refers to GMP.
