# Supplementary material for: Glucosamine Interferes With Myelopoiesis and Enhances the Immunosuppressive Activity of Myeloid-Derived Suppressor Cells
Source: Front Nutr. 2021 Nov 10;8:762363. doi: 10.3389/fnut.2021.762363 (PMC8660085; doi:10.3389/fnut.2021.762363)
Supplement: Supplementary file 3 [file Table_3.pdf]

Supplementary Table 3. The antibodies used for characterization of mouse myeloid cells

Specificity	Fluorochrome	Clone	Company	Expression
CD45	APC	30-F11	BD Bioscience	+
CD11b	BB700	M1/70	BD Bioscience	+
Gr-1	PE	RB6-8C5	BD Bioscience	+
