# Supplementary material for: Glucosamine Interferes With Myelopoiesis and Enhances the Immunosuppressive Activity of Myeloid-Derived Suppressor Cells
Source: Front Nutr. 2021 Nov 10;8:762363. doi: 10.3389/fnut.2021.762363 (PMC8660085; doi:10.3389/fnut.2021.762363)
Supplement: Supplementary file 4 [file Table_4.pdf]

Supplementary Table 4. The antibodies used for characterization of mouse MDSCs

Specificity	Fluorochrome	Clone	Company	Expression
CD45	APC	30-F11	BD Bioscience	＋
CD11b	BB700	M1/70	BD Bioscience	＋
Ly-6C	BV421	AL-22	BD Bioscience	Low/High [a]
Ly-6G	PE	1A8	BD Bioscience	－/＋ [a]

[a] Ly-6C^{Low} and Ly-6G^{+} refers to PMN-MDSC, while Ly-6C^{High} and Ly-6G^{-} refers to M-MDSC.
