# Supplementary material for: Glucosamine Interferes With Myelopoiesis and Enhances the Immunosuppressive Activity of Myeloid-Derived Suppressor Cells
Source: Front Nutr. 2021 Nov 10;8:762363. doi: 10.3389/fnut.2021.762363 (PMC8660085; doi:10.3389/fnut.2021.762363)
Supplement: Supplementary file 5 [file Table_5.pdf]

Supplementary Table 5. The antibodies used for characterization of phosphorylated ERK1/2 and STAT3 in mouse myeloid cells

Specificity	Fluorochrome	Clone	Company	Expression
CD11b	APC	M1/70	BD Bioscience	+
Gr-1	PerCP-Cy™5.5	RB6-8C5	BD Bioscience	+
ERK1/2-pT202/pY204	Alexa Fluor® 488	20A	BD Bioscience	+
Stat3-pY705	PE	4/P-STAT3	BD Bioscience	+
