# Supplementary material for: Glucosamine Interferes With Myelopoiesis and Enhances the Immunosuppressive Activity of Myeloid-Derived Suppressor Cells
Source: Front Nutr. 2021 Nov 10;8:762363. doi: 10.3389/fnut.2021.762363 (PMC8660085; doi:10.3389/fnut.2021.762363)
Supplement: Supplementary file 6 [file Table_6.pdf]

Supplementary Table 6. The antibodies used for characterization of mouse T cells

Specificity	Fluorochrome	Clone	Company	Expression
Thy1.2	APC	53-2.1	eBioscience	+
CD8	PE-Cy7	53-6.7	BD Bioscience	+
